# Evaluating treatment efficacy for Iliotibial Band Syndrome: A comparison of combined foam rolling and stretching with strengthening exercises versus strengthening alone in athletes

**DOI:** 10.1371/journal.pone.0360760

**Published:** 2026-10-27

**Authors:** Aynollah Naderi, Shanaz Azizi

**Affiliations:** 1 School of Sport Sciences, Shahrood University of Technology, Shahrood, Semnan, Iran; 2 Department of Sport Science, University College of Omran and Tosseeh, Hamadan, Iran; Universidade de Aveiro Escola Superior de Saude de Aveiro, PORTUGAL

## Abstract

Background

Iliotibial Band Syndrome (ITBS) lacks effective conservative treatment protocols, often relying on expert opinion without addressing individual factors. This study compared the effectiveness of integrating foam rolling and stretching exercises with strengthening routines versus strengthening exercises alone in athletes with ITBS. Twenty athletes with confirmed ITBS (11 males, 9 females; mean age: 33.6 ± 9.5 years) were randomized into two groups: the Integrated Training Group (ITG, n = 10) and the Strengthening-Focused Group (SFG, n = 10). Both underwent 24 sessions over eight weeks intervention, lasting 25–30 minutes per session. Outcomes included a primary measure of pain intensity (Visual Analog Scale) and secondary measures of lower extremity function (LEFS), hip abductor strength, iliotibial band flexibility, and quality of life (SF-36), were measured pre- and post-intervention. The ITG showed significantly greater improvements in pain reduction (79.7% vs. 81.0% reduction, *p* = 0.02), lower extremity function (25.7% vs. 21% improvement, *p* = 0.02), iliotibial band flexibility (59.4% vs. 1.7% improvement, *p* = 0.001), and overall QoL (19.9% vs. 12.6% improvement, *p* = 0.006) compared to the SFG. However, no significant differences were noted in hip abductor strength or physical and psychological QoL components (*p* > 0.05). Based on the sample examined, integrating foam rolling and stretching with strengthening exercises versus strengthening exercises alone resulted in greater improvements in pain, lower extremity physical function, iliotibial band flexibility, and overall QoL. However, additional studies with larger sample sizes and in other populations are needed before definitive conclusions can be reached.

## Introduction

Iliotibial Band Syndrome (ITBS) is a prevalent overuse injury affecting athletes, particularly runners and cyclists. Its prevalence ranges from approximately 16% among runners to 15–24% among cyclists [1,2]. ITBS arises from repetitive mechanical stress on the iliotibial band (ITB), leading to pain near the lateral epicondyle of the femur due to compressive and shearing forces during knee flexion and extension [3,4]. As the condition progresses, athletes often experience pain both during and after activity, significantly impacting their performance and overall quality of life (QoL) [5]. With the increasing participation in endurance sports, it is crucial to understand and address ITBS to maintain athletic performance and prevent long-term disability.

Despite extensive research on ITBS, a consensus on effective conservative treatments remains elusive. Current guidelines often rely more on expert opinion than solid empirical evidence [6,7], resulting in suboptimal recovery outcomes; only 44% of patients achieve full recovery within 2–6 weeks [8]. This inconsistency in treatment approaches, as highlighted by Ellis, Hing (2) reveals a critical information gap regarding effective protocols tailored to the diverse presentations of ITBS, particularly across varying severity levels [3]. Addressing this gap is essential, as there is a growing need to develop interventions specifically targeting the biomechanical and muscular deficiencies contributing to the syndrome.

To understand these deficiencies better, we must consider the risk factors associated with ITBS. Abnormal biomechanics—such as excessive hip adduction, internal rotation, and varus knee moments—coupled with muscle weakness or tightness, are significant contributors to the condition [9,10]. Additionally, increased ITB stiffness, often observed in affected individuals, exacerbates symptoms by elevating stress on the band and increasing pressure on the femoral condyle [11,12]. While rehabilitation strategies predominantly focus on hip abductor strengthening, which has shown effectiveness in alleviating symptoms [3,13], the potential benefits of stretching and myofascial release remain underexplored in clinical settings [7]. Recent evidence suggests that integrating these modalities with strengthening exercises could improve treatment outcomes by reducing pain and enhancing ITB flexibility [14,15]. Nevertheless, the specific contributions of stretching and myofascial release within comprehensive treatment strategies for ITBS require further investigation.

In light of these considerations, this study aims to investigate whether the addition of foam rolling and stretching to a traditional strengthening program results in superior outcomes regarding pain reduction, ITB flexibility, lower extremity function, and quality of life in athletes with ITBS. Specifically, we hypothesize that integrating foam rolling and stretching will lead to significantly greater improvements in pain, functional metrics, and health-related quality of life compared to strengthening exercises alone. By addressing the heterogeneous nature of ITBS and examining these combined interventions, this research seeks to provide clearer, evidence-based guidelines for effective treatment strategies in managing this prevalent injury.

## Methods

### Study design

This study was a randomized clinical trial that compared the effects of an 8-week intervention combining foam rolling and stretching with strengthening exercises against strengthening exercises alone (Fig 1). The study assessed pain intensity, lower extremity function, iliotibial band flexibility, hip abductor muscle strength, and quality of life (QoL), with evaluations conducted at baseline (pre-test) and at the end of the intervention (post-test).

**Fig 1:**
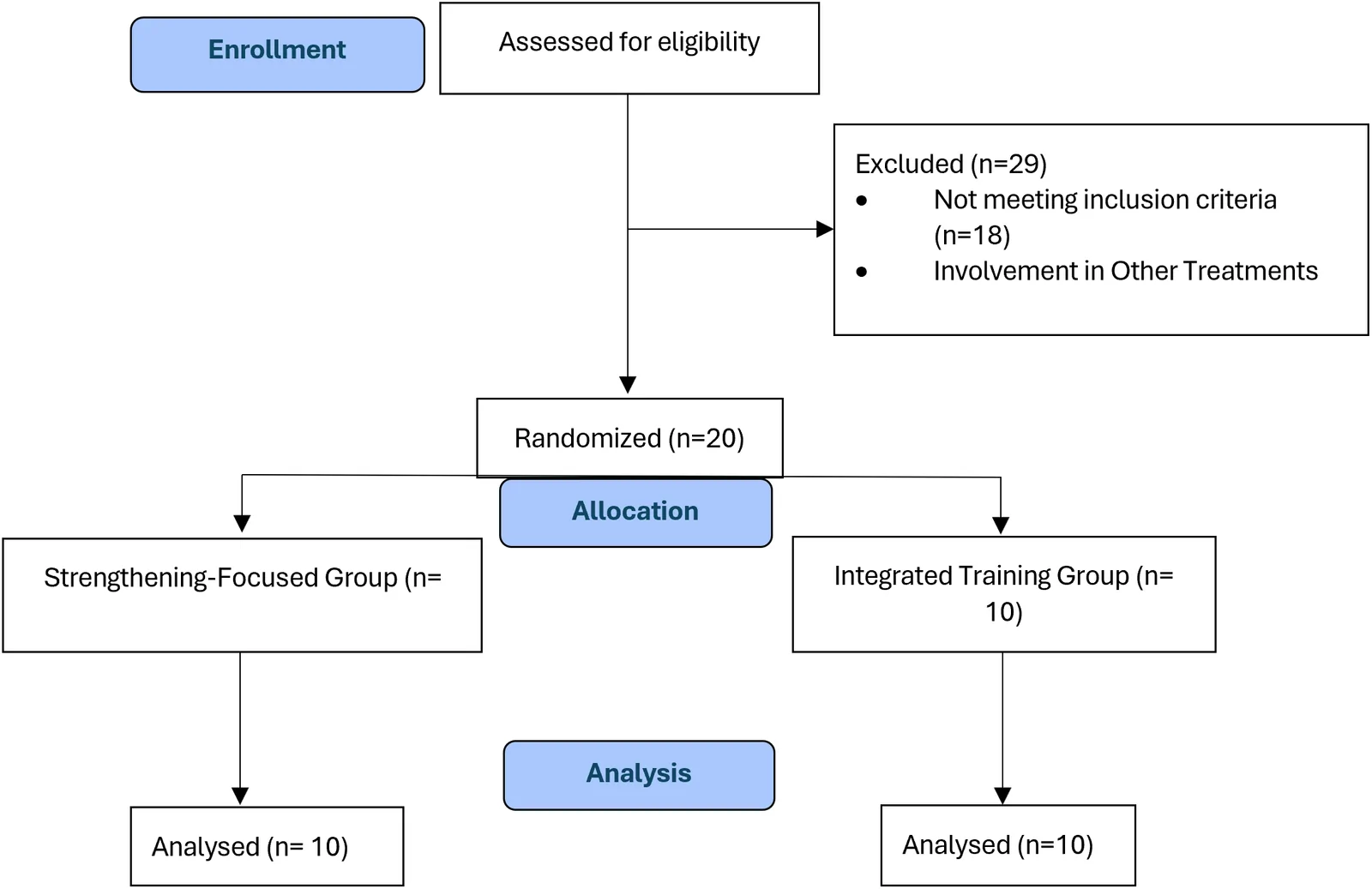
CONSORT flow diagram. Flow-chart showing inclusion, randomization and participation throughout the study.

### Participants

Sample size calculation was performed by the corresponding author (A.N.) in consultation with a biostatistician, using G*Power software (version 3.1.9.7) based on an α = 0.05, 85% power, and a conservative effect size estimate (d = 0.70) for pain intensity, as indicated byFriede, Innerhofer (7). Although no formal oversampling was performed, this conservative effect size was selected to provide adequate statistical power even in the event of minor attrition. In practice, however, all 20 randomized participants completed the study with no dropouts. These participants were prospectively recruited between December 2024 and March 2025 through presentations at community events, sports clubs, and physiotherapy centers in Sanandaj, Iran, based on established inclusion and exclusion criteria (Table 1).

**Table 1:** Inclusion and exclusion criteria.

Inclusion criteria	Exclusion criteria
Localized pain over the lateral epicondyle.	Symptoms of other knee pathologies.
Pain exacerbated by downhill running.	History of knee surgery or trauma on the affected side.
Reproducible pain during the Noble compression test [32]	Current treatment with ITB stretching or foam rolling for ITBS.
Significant discomfort at approximately 30 degrees of knee flexion.	Voluntary withdrawal from the study.
Positive results on the modified Thomas and Ober tests indicating ITB tightness [21,22]	Missing more than two consecutive sessions or a total of over five training sessions.

Both male and female athletes meeting the inclusion criteria were eligible for participation. The final sample comprised 11 males and 9 females, balanced across groups through block randomization. Written informed consent was obtained from all participants prior to enrollment. The study adhered to ethical guidelines, and confidentiality was maintained throughout. Participants were informed of their right to withdraw at any time and were offered access to the study results upon request. The study was approved by the Ethics Committee of Shahrood University of Technology (IR.SHAHROODUT.REC.1403.037) on November 12, 2024 and registered in the University Hospital Medical Information Network Clinical Trial Registry (UMIN000055882) on November 30, 2024, in accordance with the Declaration of Helsinki.”

### Randomization

After completing baseline testing, participants were randomly assigned to either the ITG or SFG. This randomization utilized a closed envelope technique with a 1:1 allocation ratio, ensuring gender balance through block randomization with block sizes of 2 and 4. These specific block sizes were selected to balance small sample sizes effectively and reduce assignment predictability. To maintain allocation concealment, group assignments remained concealed from investigators, laboratory specialists, and data analysts, facilitated by using sequentially numbered, opaque, tamper-evident sealed envelopes. An independent administrator, who was not involved in other aspects of the study, managed these envelopes, which were opened only after participants had completed all baseline assessments, thereby safeguarding the integrity of the randomization process. The CONSORT flow diagram is shown in Fig 1.

### Protocol adherence statement

No post-hoc modifications were made to the a priori study protocol after registration in the University Hospital Medical Information Network Clinical Trial Registry (UMIN000055882) and approval by the Ethics Committee of Shahrood University of Technology. All outcome measures, statistical analyses, and intervention procedures were conducted exactly as pre-specified in the registered protocol.

### Outcome measurements

The primary outcome was pain intensity measured by the 11-point Visual Analog Scale (VAS). Secondary outcomes included lower extremity function (Lower Extremity Functional Scale), hip abductor muscle strength (handheld dynamometry), iliotibial band flexibility (Ober test), and health-related quality of life (SF-36).

After selecting the study participants and completing the consent form, demographic information about the patients was collected, including age, height, weight, gender, injury history, symptomatic limb, and sporting experience. Pain intensity was measured using an 11-point Visual Analog Scale (VAS), with 0 indicating “no pain” and 10 representing “the worst pain imaginable.” Participants reported their current pain level, along with the worst and least pain experienced in the past 24 hours, and the average score was used to determine their pain level for the previous day [2]. Lower extremity function was evaluated using the Lower Extremity Functional Scale (LEFS), which contains 20 questions about an individual's ability to perform daily tasks. Each question is scored on a 5-point Likert scale (0–4), with the total score ranging from 0 to 80; a higher score indicates better lower extremity function [16]. Quality of life (QoL) was evaluated using the validated Persian version of the SF-36, which yields two summary scores—Physical (PCS) and Mental (MCS)—as well as a global QoL score calculated as the average of PCS and MCS [17,18]. All scores range from 0 to 100, with higher values reflecting better health-related QoL. The SF-36 is a reliable and responsive instrument for assessing QoL in individuals with lower-limb musculoskeletal disorders, making it suitable for use in this study [18]. The Global Rating of Change Scale (GRCS) was used to assess athletes’ perceived improvement or deterioration over time, specifically after an 8-week intervention. Participants rated their overall condition change since the intervention began, using a 15-point scale to measure perceived therapeutic effects [19]. GRCS was administered only at post-test, as it is a retrospective measure asking participants to rate their perceived change in overall condition since the start of the intervention [19]. The maximum isometric strength of the hip abductor muscles was measured using a handheld digital dynamometer, with values recorded in Newtons. Participants were positioned lying on their side, with the leg being tested on top and aligned horizontally, while the lower leg was flexed at 90 degrees at the hip and knee. They performed a maximum isometric contraction against the dynamometer for 3 seconds, followed by a 30-second rest. This process was repeated three times, and the average of the three measurements was normalized to body mass, representing the participant's strength level [20]. The flexibility of the ITB was evaluated using the Roussel and Band technique and the Ober test. Participants lay on their side with their lower limb's hip and knee flexed to 45 and 90 degrees for stabilization. An examiner applied downward pressure on the upper hip while passively moving the upper limb into abduction and extension. Participants relaxed their lower limb muscles to allow the upper limb to drop (adduction of the hip), while the examiner prevented any unwanted movements. The degree of hip adduction was measured using a goniometer, with positive values recorded if the thigh fell below horizontal and negative values if it rose above. Each measurement was taken three times, and the average was recorded as the individual's flexibility status [21,22].

### Interventions

Participants in both groups followed an initial four-week recovery phase where running and cycling were restricted, and the use of pain relievers or anti-inflammatory medications was limited to acetaminophen, taken only as needed. Ice massage was applied to the affected area twice daily for 10 minutes during the first two weeks, with participants allowed to adjust the duration and frequency based on individual tolerance. All participants participated in a structured exercise program conducted three times a week, with each session lasting 25–30 minutes. Every session included a 5-minute warm-up and a 5-minute cool-down, featuring walking, jogging in place, high knees, and flexibility exercises. The intensity of the sessions increased progressively, and heart rate targets, alongside the Borg scale, were used to monitor exertion.

**Strengthening-Focused Group (SFG):** The SFG implemented a hip abductor-focused exercise program that included various exercises such as hip abduction, clamshells, pelvic drop exercises, single-leg step-downs, forward and backward lunges, modified matrix exercises, and wall-banger exercises [7,14,15,23,24]. This program aimed to enhance lower limb stability, strength, and control, specifically targeting the hip abductor muscles (For more details, see Table 2).

**Table 2:** Intervention components delivered to each experimental group.

Exercise	Description	Target Tissue/Muscle	Assigned Group
Hip Abduction	Side-lying leg raise, keeping the top leg straight and lifting it upward.	Hip abductors, especially gluteus medius	ITG, SFG
Clamshells	Side-lying with knees bent, lifting the top knee while keeping feet together.	Gluteus medius, gluteus maximus	ITG, SFG
Pelvic Drop	Standing on one leg on a step, lowering the opposite pelvis, then lifting it back to neutral.	Gluteus medius	ITG, SFG
Single-Leg Step-Down	Stepping down from a platform with one leg, ensuring knee control.	Gluteus medius	ITG, SFG
Forward and Backward Lunges	Stepping forward or backward into a lunge position, ensuring knee alignment.	Lower limb stabilizers	ITG, SFG
Modified Matrix Exercises	Multi-directional lunges to improve stability and control in various planes of motion.	Lower limb stabilizers	ITG, SFG
Wall-Banger Exercises	Standing sideways to a wall, pushing against it with the hip to activate hip abductors.	Gluteus medius and core muscles	ITG, SFG
Standing Stretches	Stand upright, cross one leg behind the other, and lean sideways to stretch the outer thigh and hip.	Iliotibial Band (ITB)	ITG only
Side-Lying Pretzel Stretch	Lie on the side, bend the top knee, and pull the foot toward the glutes while rotating the torso backward.	ITB	ITG only
ITB Release with Foam Rolling	Lie sideways with the foam roller placed under the outer thigh, rolling from the hip to just above the knee.	ITB	ITG only

**Integrated Training Group (ITG):** Participants in the ITG followed the same strength training regimen as the SFG, supplemented with a stretching and foam rolling program that included standing stretches, side-lying Pretzel stretches, and ITB release with foam rolling [5,23,24]. Crucially, within each session, the strengthening exercises were performed first, followed immediately by the stretching and foam rolling protocol during the cool-down phase. This specific sequencing was intentionally designed to avoid any acute, temporary reductions in muscle force production that can occur with pre-exercise static stretching, thereby ensuring participants could execute the strengthening exercises with maximal effort and proper neuromuscular control, while utilizing the subsequent myofascial release and stretching to optimize tissue compliance and promote relaxation. This combined approach aimed to provide a holistic solution for improving lower limb function and reducing pain in athletes with ITBS (For more details, see Table 2).

After the four-week recovery phase, both groups followed a gradual, symptom-guided return-to-running protocol. In alignment with biomechanical evidence indicating that faster-paced running with higher cadence reduces compressive strain on the iliotibial band compared to slower jogging [25], the protocol was initiated with shorter, faster run-walk intervals. Following this initial phase, running volume was gradually increased by no more than 10% per week. Participants were instructed to reduce their training volume or stop running if pain was experienced, ensuring an appropriate stress level was applied to the tissues to promote safe and effective recovery [26].

Attendance was recorded at each session for each group. Compliance was calculated as the percentage of completed sessions out of the total 24 scheduled sessions. Participants who missed more than five sessions were excluded from the analysis, though no participants met this criterion.

### Statistical analyses

Data were analyzed using SPSS statistical software version 22 (IBM, Armonk, New York), reporting the results as means with standard deviations and mean differences with 95% confidence intervals. Data normality and homogeneity of variances were assessed using the Shapiro-Wilk and Levene’s tests, respectively. Between-group comparisons were performed using the independent t-test for age, height, weight, and body mass index; the Mann-Whitney U test for injury history and sporting experience; and the chi-square test for sex and symptomatic limb. Separate ANCOVAs were performed to compare the effects of combined and strengthening interventions on pain intensity, lower limb function, iliotibial band flexibility, hip abductor strength, and quality of life. In each model, post-test values served as the dependent variable, group assignment (ITG vs. SFG) as the fixed factor, and baseline scores for the respective outcome variable plus injury history (months). This approach was used to statistically adjust for any pre-existing between-group differences at baseline, thereby enhancing the internal validity of our between-group comparisons. A Mann-Whitney U test was conducted to compare the two groups on the Global Rating of Change Scale (GRCS), which was administered only at post-test to assess athletes’ perceived improvement or deterioration over the course of the 8-week intervention. As the GRCS is a retrospective measure asking participants to rate their overall change since the intervention began, baseline comparison was not applicable. Percentage changes from baseline were calculated for each outcome variable using the formula: [(Post-test mean − Pre-test mean) / Pre-test mean] × 100. Statistical significance was set at p < .05. To better interpret the range of training gains, Cohen’s d was calculated to express effect sizes, with values between 0.01 and 0.2 considered trivial, 0.21 to 0.50 as small, 0.51 to 0.80 as medium, and values over 0.81 representing large effects.

## Results

All 20 randomized participants completed the study protocol and were included in the final analysis. Compliance with the intervention was high in both groups: the ITG attended 96.7% ± 2.1% of sessions, and the SFG attended 95.0% ± 3.0% of sessions (p = 0.48). Table 3 presents the descriptive characteristics of the participants, including age, height, weight, body mass index (BMI), sex distribution, injury history, symptomatic limb, and sporting experience. Statistical analysis showed no significant differences between the SFG and ITG across all variables (*p* > 0.05), indicating that the groups were comparable at baseline.

**Table 3:** Descriptive information of participants’ physical characteristics.

Variables	SFG (n = 10)	ITG (n = 10)	p-value
Sex (Male/Female) *	5/5	6/4	1.00
**Age (years)** ^#^	33.1 ± 9.5	34.1 ± 9.6	0.82
**Height (cm)** ^#^	173.9 ± 6.7	174.3 ± 9.4	0.90
**Weight (kg)** ^#^	76.3 ± 15.8	78.9 ± 17.1	0.72
**Body Mass Index (kg/m²)** ^#^	25.2 ± 4.8	25.8 ± 4.4	0.75
**Injury History (months)** ^$^	11.0 ± 7.4	8.2 ± 2.9	0.29
Symptomatic Limb (Right/Left/Both) *	6/1/3	6/2/2	0.77
**Sporting Experience (years)** ^$^	6.9 ± 3.1	7.2 ± 3.2	0.80

**Note:** Mean with standard deviation (mean ± SD) or frequency were presented. ITG: Integrated Training Group, SFG: Strengthening-Focused Group. Between-group comparisons were performed using the independent t-test (#) for continuous variables with normal distribution, the Mann-Whitney U test ($) for non-normally distributed variables, and the chi-square test (*) for categorical variables.

The ANCOVA revealed a significant group effect for pain intensity, F (1, 19) = 6.4, p = .02, indicating a substantial difference between the groups at the post-test. Over the 8-week study period, the ITG experienced a remarkable 79.7% reduction in pain intensity, while the SFG showed an 81.0% decrease (Cohen’s *d* = −1.25). A significant group effect for lower limb function was also identified at post-test, F (1, 19) = 7.1, p = .02, showing that the ITG improved by 25.7%, compared to a 21% improvement in the SFG (Cohen’s *d* = 1.20). Additionally, a significant group effect for ITB flexibility was observed, F (1, 19) = 21.70, p = .001, with the ITG demonstrating a 59.4% increase, whereas the SFG showed only a 1.7% improvement (Cohen’s *d* = 0.82). There were no significant group differences for hip abductor muscle isometric strength, F (1, 19) = 3.20, *p* = .16, with a small effect size (Cohen’s *d* = 0.12). The ITG showed a 7.9% improvement, while the SFG showed a 5.7% improvement. Finally, a significant group effect for QoL was found, F (1, 19) = 9.90, p = .006. However, there were no significant differences in the physical (F (1, 19) = 3.90, p = .07, Cohen’s *d* = 1.24) or psychological (F (1, 19) = 3.20, p = .08, Cohen’s d = 0.54) components of QoL. The ITG showed a 19.9% improvement in overall QoL, compared to an 12.6% improvement in the SFG (Cohen’s d = 1.33). These findings are detailed in Table 4.

**Table 4:** Descriptive data and ANCOVA results for pain intensity in athletes with Iliotibial Band Syndrome (ITBS).

Variables	Time Point	ITG (n = 10)	SFG (n = 10)	*Mean Difference* 95% CI	Group Effect		Cohen’s d
					(f)	(p)	
**Pain Intensity (0–10 points)**	Pre-test	4.7 ± 1.6	5.1 ± 1.2	−0.3 (−1.6 to 1.03)	6.4	0.02*	−1.25
	Post-test	0.94 ± 1.0	0.94 ± 2.0	−1.1 (−1.9 to −0.3)			
**Lower Limb Function (0–80 points)**	Pre-test	60.2 ± 9.7	58.2 ± 9.6	2.0(−7.0 to 11.0)	7.1	0.02*	1.20
	Post-test	75.7 ± 3.6	70.4 ± 4.8	5.3(1.3 to 9.3)			
**Iliotibial Band Flexibility (degrees)**	Pre-test	10.1 ± 5.6	11.5 ± 6.1	−1.4(−6.9 to 4.1)	21.7	0.001*	0.82
	Post-test	16.1 ± 4.6	11.7 ± 5.6	4.4(0.4 to 8.4)			
**Hip Abductor Muscle Strength (percent of body mass)**	Pre-test	1.91 ± 0.43	1.94 ± 0.31	−0.04 (−0.4 to 0.3)	3.20	0.16	0.12
	Post-test	2.06 ± 0.36	2.05 ± 0.31	0.01 (−0.3 to 0.3)			
**Quality of Life (0–100 points)**	Pre-test	68.8 ± 4.0	67.6 ± 7.7	0.2 (−4.7 to 5.1)	9.9	0.006*	1.33
	Post-test	82.5 ± 3.3	76.1 ± 8.0	4.0 (0.2 to 8.3)			
**Physical Component of QoL** **(0–100 points)**	Pre-test	67.0 ± 4.8	65.3 ± 13.3	−0.4 (−7.5 to 6.8)	3.9	0.07	1.24
	Post-test	80.7 ± 4.5	74.1 ± 11.0	4.4 (−3.7 to 12.5)			
**Psychological Component of QoL (0–100 points)**	Pre-test	70.6 ± 4.8	69.8 ± 13.3	0.8 (−5.9 to 7.5)	3.2	0.08	0.54
	Post-test	84.4 ± 4.7	80.1 ± 6.8	3.6 (−1.9 to 9.1)			

**Note:** *. Significant difference at p < 0.05. ITG: Integrated Training Group, SFG: Strengthening-Focused Group. The presented table includes Cohen's d effect sizes for between-group comparisons. In the ANCOVA model, baseline scores for each outcome variable and injury history (months) were included as covariates to statistically control for pre-existing between-group differences at baseline.

The Mann–Whitney U test indicated that participants in the ITG tended to report more favorable perceived therapeutic effects than those in the SFG; however, this difference was not statistically significant (z = 1.24, p = 0.25; Fig 2).

**Fig 2:**
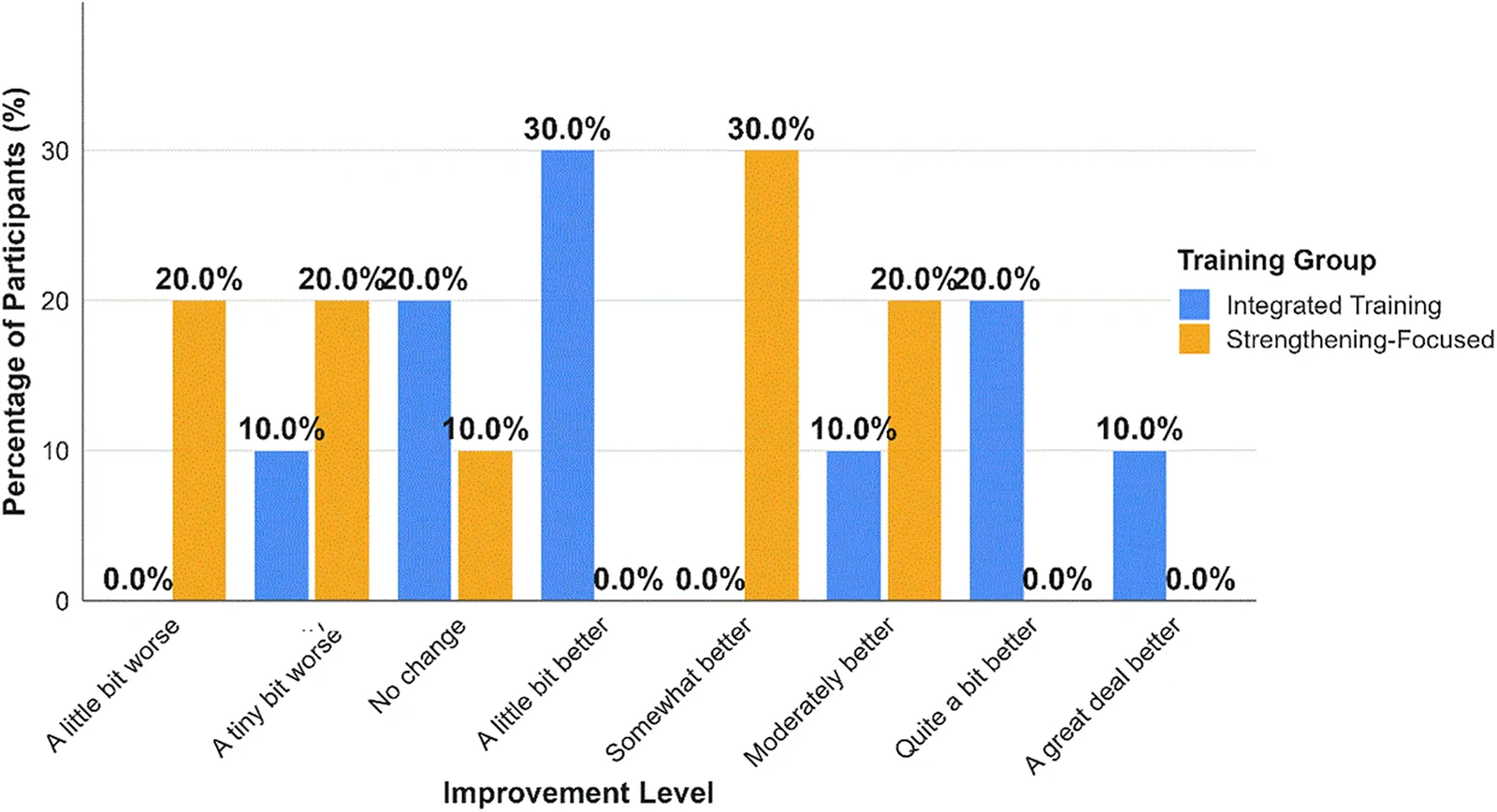
Perceived treatment effect by participants of each group at post-test.

## Discussion

The findings of this study indicate that the ITG achieved significant improvements across multiple outcomes, with large effect sizes illustrating the difference between the ITG and the SFG in the post-test results. Specifically, the ITG experienced a 79.7% reduction in pain intensity, compared to an 81.0% reduction in the SFG. In addition, lower limb function improved by 25.7% (a 15.5-point gain on the LEFS) in the ITG, versus a 21% (12.2-point) improvement in the SFG. Similarly, ITB flexibility showed a marked increase of 59.4% (a 6.0° gain in the Ober test) in the ITG, while the SFG improved by only 1.7% (0.2°). Finally, overall QoL improved by 19.9% (a 13.7-point gain on the SF-36) in the ITG, compared to a 12.6% (8.5-point) improvement in the SFG. These results strongly suggest that the combined training approach, incorporating foam rolling and stretching with strengthening exercises, is more effective than strength training alone in reducing pain, enhancing function, increasing flexibility, and improving QoL for athletes with ITBS.

A closer examination of the pain reduction data reveals the significant benefit of the combined training approach, with a Cohen’s d of −1.25. This result aligns with Friede, Innerhofer (7), whose research showed substantial pain relief in patients with ITBS following a six-week multi-modal physiotherapy program that included foam rolling and stretching. The integration of multiple intervention strategies in our study likely enhanced pain relief by addressing both physiological and psychological mechanisms associated with pain and recovery. The synergy of these approaches appears to generate a cumulative effect greater than strength training alone, supporting the hypothesis that multifaceted interventions may be more effective for pain management in ITBS.

The functional improvements observed in the ITG underscore the advantages of a multi-modal approach, with a 25.7% increase in lower limb function compared to 21% in the SFG, yielding a Cohen’s d of 1.20. This suggests that the combination of foam rolling, stretching, and strengthening exercises significantly enhances functional outcomes. These findings align with studies by Friede, Innerhofer (7) andPark, Lee (5), which demonstrated performance improvements following multi-modal interventions in individuals with ITBS.Friede, Innerhofer (7) highlighted functional gains after a structured physiotherapy program, while Park et al. noted flexibility and performance enhancements in cyclists after myofascial release. In contrast, Park, Lee (5)reported less pronounced functional improvements with strength training alone, further reinforcing the notion that a comprehensive approach integrating various techniques yields superior functional results. The enhanced pain reduction and functional improvements in the ITG can be better understood through a neurophysiological lens. The integration of foam rolling, stretching, and strengthening exercises likely accelerates recovery by improving blood flow, reducing muscle tension, and increasing flexibility. Foam rolling and stretching specifically target the release of fascial tightness and muscle tension, while strength exercises help build supportive musculature for recovery. Additionally, neurophysiological mechanisms like gate control theory likely inhibit pain perception, providing immediate relief. The variety of exercises may also enhance adherence and motivation, leading to better overall outcomes for athletes.

Similar to the findings for pain and function, the significant improvement in ITB flexibility (59.4% in the ITG versus 1.7% in the SFG) suggests that a multi-modal intervention provides distinct advantages over strength training alone. The Cohen’s d of 0.82 further highlights the effectiveness of the combined approach. Foam rolling and stretching, in particular, are known to alleviate muscle tightness, improve tissue elasticity, and reduce fascial restrictions, all of which contribute to enhanced flexibility. In contrast, strength training alone does not directly address flexibility, limiting its potential to improve ITB flexibility. These results are consistent withPark, Lee (5), which showed significant gains in flexibility and pain reduction in cyclists after myofascial release.

Furthermore, it is important to interpret the improvements in the Ober test with biomechanical nuance. Recent cadaveric evidence indicates that the Ober test primarily measures the compliance of proximal hip structures (e.g., gluteus medius, minimus, and hip capsule) and the continuous myofascial chain, rather than the iliotibial band in isolation [27]. Therefore, the significant improvement in Ober test scores observed in the ITG likely reflects a synergistic reduction in tension across this entire proximal-to-distal complex. The addition of targeted stretching and foam rolling to the strengthening protocol may have more effectively decreased the viscoelastic resistance and neurophysiological tone of the tensor fasciae latae and associated proximal structures than strengthening exercises alone.

Finally, the observed improvements in overall QoL further highlight the benefits of the combined intervention, with a 19.9% increase in the ITG compared to 12.6% in the SFG (Cohen’s d = 1.33). However, it is noteworthy that no significant changes were found in the physical or psychological components of QoL, raising important considerations for interpreting these outcomes. The overall increase in QoL may stem from factors beyond physical or psychological health, such as enhanced social interaction, structured program engagement, and increased motivation. These aspects could improve participants’ general well-being even without measurable shifts in specific health domains. The absence of significant changes in the physical and psychological components might also be due to the study’s short duration, which may have been insufficient to capture longer-term effects. Additionally, the SF-36 scoring method, which averages the physical and psychological components, might obscure smaller, incremental improvements that could become significant over time. Therefore, while the overall QoL score reflects positive changes, these results should be interpreted cautiously, acknowledging the complex nature of QoL and the potential influence of non-health-related factors.

The clinical relevance of our findings is supported by comparison with established Minimal Clinically Important Differences (MCIDs). For pain intensity, a reduction of ≥30% or ≥1.5 points on a 0–10 VAS is considered clinically meaningful [28,29]; in our study, the ITG demonstrated a 79.7% reduction and the SFG a 81.0% reduction, both exceeding this threshold. Similarly, for lower extremity function, an improvement of ≥9 points on the LEFS represents a meaningful clinical change [30,31], with the ITG improving by 15.5 points and the SFG by 12.2 points, indicating that both interventions yielded clinically relevant benefits, though the combined approach provided superior magnitude. Regarding iliotibial band flexibility, while no universally accepted MCID has been established, Ferber, Kendall (22)provided normative and critical criteria using the Ober test, suggesting that changes ≥5° may represent functionally meaningful improvements in athletic populations; notably, the ITG demonstrated a 6.0° increase (59.4% improvement), exceeding this suggested threshold, whereas the SFG showed only a 0.2° increase (1.7% improvement), indicating that the combined intervention provided clinically relevant gains in flexibility that strengthening alone did not achieve. Finally, for hip abductor strength measured via handheld dynamometry, Arnold, Warkentin (20)demonstrated excellent reliability (ICC = 0.80–0.96) but did not establish a specific MCID; although the ITG (7.9%) and SFG (5.7%) showed modest gains that did not reach statistical significance (p = 0.16), the observed improvements suggest both interventions contributed to strength development, with the combined approach yielding slightly greater magnitude, a finding we interpret with appropriate caution given the absence of validated MCIDs for this outcome in ITBS populations.

### Strength and limitations

This study offers several notable strengths. Firstly, the randomized clinical trial design provides a robust framework for evaluating causal relationships between the interventions and outcomes. Secondly, the inclusion of both subjective (e.g., pain, QoL) and objective (e.g., flexibility, strength) outcome measures allows for a multidimensional assessment of recovery. Thirdly, the use of ANCOVA adjusted for baseline values and injury history strengthens the internal validity by controlling for potential confounders. Lastly, the standardized 8-week protocol with monitored adherence enhances the reproducibility and clinical applicability of the findings.

One limitation of this study is the relatively small sample size, which may reduce the generalizability of the findings and limit the statistical power to detect significant differences in secondary outcomes such as muscle strength and flexibility. Additionally, the 8-week intervention period may not have been long enough to observe sustained changes in these variables, particularly muscle strength and flexibility adaptations, which could take longer to develop. Furthermore, although the handheld dynamometry protocol employed in this study followed a standardized procedure with three repeated trials averaged per session (ICC = 0.80–0.96) [20], all hip abductor strength measurements were performed by a single examiner. Formal intra-rater reliability testing across separate testing days was not conducted, which may limit the generalizability of the strength data. Future studies are encouraged to incorporate multi-examiner designs or formal intra- and inter-rater reliability assessments to further validate dynamometric measurements in ITBS populations. Additionally, while the Ober test is a widely used clinical measure, cadaveric studies suggest it may reflect the compliance of proximal hip structures and the broader myofascial chain rather than the ITB in isolation [27]. Future research should consider incorporating objective imaging modalities, such as shear-wave elastography, to directly quantify the stiffness of specific tissues within this complex. The absence of participant or assessor blinding poses a risk of bias, especially in subjective outcomes like pain intensity and perceived treatment improvement. The reliance on self-reported data for lower limb function introduces the potential for bias. To mitigate this, future studies could incorporate objective assessments such as 3D motion analysis, wearable sensors (e.g., accelerometers, gyroscopes, and inertial measurement units), isokinetic dynamometry, electromyography (EMG), or force plates, providing a more reliable and comprehensive evaluation of lower limb function. Furthermore, the lack of long-term follow-up limits our ability to assess the durability of the intervention effects.

### Practical implications

The practical implications of this study are crucial for managing ITBS in athletes. The findings indicate that integrating foam rolling and stretching with strengthening exercises results in greater improvements in pain reduction, lower limb function, flexibility, and QoL compared to strength training alone. This combined approach can be easily adopted by sports coaches, physical therapists, and rehabilitation specialists to optimize recovery and enhance athletic performance. Furthermore, the significant improvements in pain and function suggest that these methods can be incorporated into routine injury prevention programs, potentially reducing the risk of future ITBS occurrences and supporting long-term athlete health.

Future directions should explore the long-term effects of this combined approach on the recurrence of ITBS, including how it impacts athletic performance in various sports. Further research could also investigate the optimal frequency and duration of these interventions to maximize benefits. Clinical trials and studies with larger sample sizes could provide additional evidence to support the widespread adoption of these practices in sports medicine.

## Conclusion

The findings of our study highlight the effectiveness of combining foam rolling and stretching exercises with strengthening routines for athletes with ITBS. Athletes who engaged in this combined training program experienced greater improvements in pain relief, lower limb function, flexibility, and overall QoL compared to those who focused solely on strengthening exercises. These results suggest that incorporating foam rolling and stretching into rehabilitation protocols can enhance recovery, helping athletes manage ITBS more effectively and return to their activities with improved function and well-being.

## Supporting information

S1 File Ethics committee protocol. (DOCX)
