# Supplementary material for: Evaluating treatment efficacy for Iliotibial Band Syndrome: A comparison of combined foam rolling and stretching with strengthening exercises versus strengthening alone in athletes
Source: PLoS One. 2026 Oct 27;21(10):e0360760. doi: 10.1371/journal.pone.0360760 (PMC13712515; doi:10.1371/journal.pone.0360760)
Supplement: Supplementary file 1 [file pone.0360760.s001.docx]

**Dear Researcher,**

The Ethics Committee for Biomedical Research at Shahrood University of Technology reviews research proposals in accordance with the ethical guidelines of the Ministry of Science, Research and Technology and the Ministry of Health and Medical Education. This review focuses on compliance with research ethics principles and the protection of human or animal participants.

Researchers are kindly requested to carefully study the relevant guidelines and complete this form in a way that assures the Ethics Committee members of full adherence to ethical principles in the proposed research.

**Research Identification Form**

**Research Title**

Persian:
**مقایسه تاثیر رول فوم، تمرینات کششی و تقویتی با تمرینات تقویتی تنها در مدیریت ورزشکاران مبتلا به سندرم نوار ایلئوتیبیال**

English:
Comparison of the effect of foam roll, stretching and strengthening exercises with strengthening exercises only in the management of athletes with iliotibial band syndrome

**• Scientific Field Related to the Research**

☐Sport Management

☐ Exercise Physiology

☐ Sport Psychology

☐ Motor Behavior

☐ Biomechanics and Sport Technology

☐ Sociology, Philosophy, and History of Sport

☐ Sport Tourism and Marketing

☑ Sport Injury and Corrective Exercises

☐ Sport Communication and Media

**• Research Format**

☑ Thesis / Dissertation

☐ Organizational Research Project

☐ Independent Student Research Project

☐ Invention

**Name of the Supporting University or Organization**

University College of Omran and Tosseeh

**Place of Research Implementation**

University College of Omran and Tosseeh

**A) Full Details of the Principal Researcher (Supervisor or Project Leader)**

- **Full Name (Persian):** عین اله نادری
- **Full Name (English):** Aynollah Naderi
- **National ID Number:** 2370751800
- **Affiliation / Workplace:** Shahrood University of Technology
- **Highest Academic Degree:** PhD
- **Field of Specialization:** Physical Education and Sport Sciences
- **Postal Address:** Faculty of Physical Education and Sport Sciences, Shahrood University of Technology
- **Contact Number:** +98 917 721 7462
- **Email Address:** Ay.naderi@yahoo.com

**B) Details of Other Researchers (Co-Supervisor, Student, or Research Collaborators)**

| **Full Name**  **(Persian)** | **Full Name (English)** | **National ID** | **Position** | **Mobile Phone** | **Email** |
| --- | --- | --- | --- | --- | --- |
| شاناز عزیزی | Shanaz Azizi | — | MSc Student | +98 918 416 6818 | aa99az88@gmail.com |

**C) Applicant for the Ethics Approval Certificate From**

☐ Ministry of Science, Research and Technology

☑Ministry of Health and Medical Education

**Note:** Obtaining an ethics approval code from the Ministry of Health usually requires a longer review period.

**Ethics Proposal Text**

**Important Notice:**

To facilitate and expedite the review process and the issuance of the ethics approval certificate, please carefully read the instructions provided under each section before completing the form. Then, replace the instructional text with the appropriate content for each section. Adherence to the prescribed framework and structure of the proposal is a necessary requirement for entry into the ethical review process.

1. **Introduction**

The knee is the most common site of overuse injuries in the running population, with iliotibial band syndrome (ITBS) being one of the most prevalent conditions[1, 2]. ITBS is the second most common cause of knee pain in runners, affecting approximately 16% of runners[3] and 15–24% of cyclists [4].

The iliotibial band (ITB) is a thickened lateral portion of the fascia lata, composed primarily of longitudinally oriented collagen fiber bundles. The ITB originates from the tensor fasciae latae (TFL) and gluteus maximus muscles and inserts onto the lateral femoral epicondyle and the anterolateral aspect of the tibia[5]. During running and cycling, the ITB is subjected to substantial mechanical stress through the muscles attached to it, which may lead to overuse injuries in the distal portion of the ITB. The primary source of injury is repetitive compressive and shear stress generated by the movement of the ITB over the lateral femoral epicondyle during knee flexion and extension[6].

Typically, pain associated with ITBS occurs proximal to the lateral joint line, near the lateral femoral epicondyle. To date, two overlapping theories have been proposed to explain the pathophysiology of ITBS[2].

Proponents of the first theory believe that ITBS results from enthesopathy and compression of fat tissue, blood vessels, nerves, and Pacinian corpuscles during knee flexion at the insertion site of the iliotibial band tendon[7]. The second theory, which reinforces the initial hypothesis with a friction and impingement model, suggests that the posterior fibers of the iliotibial band slide in an anterior–posterior direction over the lateral femoral condyle, leading to the development of ITBS[8]. Friction is thought to be greatest at approximately 30 degrees of knee flexion.

Pain associated with ITBS occurs in response to excessive physical activity involving cyclical lower-limb movements such as running or cycling. The pain is exacerbated by eccentric contraction of the tensor fasciae latae and gluteus maximus muscles, particularly during downhill running or descending stairs, as these muscles act to decelerate the body[9] .

Although ITBS was initially considered a friction-based syndrome, more recent evidence suggests that it is primarily caused by excessive tension within the iliotibial band, resulting in chronic compression of the underlying tissues (such as fat pads or bursae) and subsequent inflammation and pain[10]. In the early stages, pain often appears after completion of activities involving repetitive knee flexion–extension. However, as the condition progresses, symptoms may occur earlier during physical activity and even at rest. In addition, downhill running, running on uneven surfaces, using a longer stride length, and prolonged sitting with the knee in a flexed position often exacerbate pain. These symptoms negatively affect not only knee function but also the physical and psychological components of quality of life. Therefore, the prevention and treatment of this injury are of increasing importance in athletic populations.

Despite numerous efforts to understand the mechanisms underlying ITBS, the optimal conservative treatment for its management remains unclear. Consequently, recommendations and guidelines for the conservative management of ITBS are often largely based on expert opinion rather than strong empirical evidence[11, 12]. Ellis et al., in a review study, highlighted the irrationality of many treatment methods used by clinicians, noting that these approaches were frequently unsupported by evidence or based on minimal evidence[4].

Therefore, it is not surprising that the effectiveness of conservative treatments in this area has been discouraging, with only 44% of patients achieving complete recovery after 2 to 6 weeks of conservative management[13]. Given that individuals with ITBS represent a heterogeneous patient population and that different subgroups may respond differently to specific treatments, recent studies have emphasized the importance of designing targeted interventions tailored to distinct ITBS subgroups[6].

Abnormal lower-limb biomechanics, as well as weakness and tightness of lower-limb muscles, are among the risk factors for developing ITBS[14, 15]. In this context, stiffness or shortening of the ITB is described as a common objective sign in patients with ITBS, and addressing it is a primary goal of therapeutic exercise [14-16]. For example, excessive hip adduction and internal rotation, internal tibial rotation, and knee varus moments can increase tensile stress on the ITB, thereby elevating pressure on the lateral femoral condyle[14-16]. ITB tightness can also amplify compressive forces on the underlying tissues, leading to the manifestation of ITBS symptoms[14-16].

Although ITBS is considered a multifactorial condition with no single definitive treatment, the core components of its rehabilitation generally include rest or activity modification, ITB stretching, and strengthening of the hip abductors. In this regard, Farid et al. recently concluded that individualized strengthening of the hip abductor muscles is a key component of ITBS rehabilitation, while they placed less emphasis on the specific methods used for ITB stretching and release[6, 17].

Stretching is one of the recommended methods in review studies[18]; however, it has not been thoroughly investigated in clinical practice and, importantly, has never been compared against a valid control intervention. Nonetheless, there is evidence suggesting that stretching and myofascial release modalities may be beneficial for individuals with ITBS in terms of pain reduction, increased hip range of motion, and improved functional performance[18, 19].

Studies that have evaluated the isolated effects of these modalities in individuals with ITBS have primarily focused on their acute effects. Evidence from long-term interventional studies indicates that using stretching and release modalities as part of a multifaceted treatment approach can be beneficial for individuals with ITBS [9]. However, it remains unclear how much these modalities contribute to treatment outcomes when incorporated into a comprehensive rehabilitation program for ITBS.

Recent evidence also suggests that patients with ITBS do not necessarily have a stiffer ITB, and an increase (rather than a decrease) in ITB stiffness may even be associated with favorable treatment outcomes. Nevertheless, the role of isolated exercise modalities (e.g., stretching, myofascial release) has not been extensively studied and remains uncertain. Therefore, the present study aims to compare the effects of foam rolling and stretching combined with strengthening exercises versus strengthening exercises alone in the management of athletes with iliotibial band syndrome (ITBS).

**2. Research Objectives or Hypotheses**

**Research Objectives**

**General Objective**

The general aim of the present study is to compare the effectiveness of foam rolling and stretching exercises combined with strengthening exercises versus strengthening exercises alone in the management of athletes with iliotibial band syndrome (ITBS).

**Specific Objectives**

1. To compare the effects of foam rolling and stretching exercises combined with strengthening exercises versus strengthening exercises alone on pain intensity in athletes with iliotibial band syndrome.
2. To compare the effects of foam rolling and stretching exercises combined with strengthening exercises versus strengthening exercises alone on lower limb function in athletes with iliotibial band syndrome.
3. To compare the effects of foam rolling and stretching exercises combined with strengthening exercises versus strengthening exercises alone on athletes’ perceived therapeutic effects in those with iliotibial band syndrome.
4. To compare the effects of foam rolling and stretching exercises combined with strengthening exercises versus strengthening exercises alone on hip abductor muscle strength in athletes with iliotibial band syndrome.
5. To compare the effects of foam rolling and stretching exercises combined with strengthening exercises versus strengthening exercises alone on hip abductor muscle flexibility in athletes with iliotibial band syndrome.
6. To compare the effects of foam rolling and stretching exercises combined with strengthening exercises versus strengthening exercises alone on quality of life in athletes with iliotibial band syndrome.

**3. Methodology**

**Type of Research**

Considering the nature of the topic, the present study is applied research. Based on the design, it is a clinical trial conducted using a pretest–posttest format with a control group over a period of 8 weeks in Sanandaj city.

**Study Population and Sampling Method**

The study population consists of athletes with iliotibial band syndrome (ITBS) in Sanandaj city. The sample size was calculated based on the results of the study by Cruz et al.[20] using G*Power software version 3. The calculation was performed with an effect size of f = 0.28, a significance level of α = 0.05, and a statistical power of 85% (β). Accordingly, 20 participants were required for the present study. These individuals will be purposively selected from the study population through screening based on the inclusion and exclusion criteria.

**Inclusion and Exclusion Criteria**

Participants were diagnosed by an orthopedic specialist based on clinical history, disease manifestations, and physical examination.

**Inclusion Criteria:**

1. Pain and localized tenderness over the lateral femoral epicondyle.
2. Pain exacerbated by running, particularly downhill.
3. Pain elicited by the Noble compression test.
4. Pain during knee flexion and extension when pressure is applied over the lateral femoral condyle, with maximal pain occurring at approximately 30° of knee flexion.
5. Positive modified Thomas and Ober tests confirming ITB tightness.

**Exclusion Criteria:**

Participants showing symptoms or signs of any other knee pathology, such as meniscal injury, arthritis, ligament injury, or popliteal tendinitis, were excluded. Other knee pathologies were also ruled out by the same orthopedic specialist based on clinical history, disease manifestations, and physical examination. Participants with a history of knee surgery or trauma on the affected side, or those currently using ITB stretching complexes or foam rolling for ITBS treatment, were excluded. Participants who voluntarily chose to withdraw were also excluded. Additionally, participants who missed more than two consecutive sessions or more than five total training sessions were removed from the study.

**Dependent Variables of the Study**

Pain Intensity

Pain intensity was assessed using an 11-point Visual Analog Scale (VAS). A score of 0 on the left side represented “no pain,” and a score of 10 on the right side represented “the worst pain imaginable.” Previous studies have demonstrated that the VAS has good validity and reliability[21]. Participants rated their current pain, as well as the worst and least pain experienced during the previous 24 hours, on the scale. The mean of these scores was considered the participant’s pain level for the preceding day.

Quality of Life

Health-related quality of life was assessed using the SF-36 questionnaire. This instrument evaluates quality of life across eight health domains, including physical function, role limitations due to physical problems, general health , role limitations due to emotional problems, bodily pain, social functioning, vitality, and mental health. In the SF-36 scale, lower scores indicate poorer health-related quality of life. The validity and reliability of both the original version and the Persian version of this questionnaire have previously been confirmed [22].

Lower Limb Function

Lower limb function was assessed using the Lower Extremity Functional Scale (LEFS), which consists of 20 items evaluating an individual’s ability to perform daily activities. Each item is scored on a 5-point Likert scale ranging from 0 to 4. The total score is calculated by summing the item scores, yielding a range from 0 to 80. In this scale, higher scores indicate better lower limb function[23].

Assessment of Perceived Treatment Effect

To evaluate the degree of improvement or deterioration in athletes’ condition over time, the Global Rating of Change Scale (GRCS) was used. This scale is applied to determine the effect of an intervention or to assess the clinical course of an injury. The GRC was completed by all participants at the end of the intervention period (week 8, final assessment). Participants were asked to independently rate the overall change in their condition from the start of the intervention to the present on the scale. A 15-point scale was used to assess the perceived treatment effect. This 15-point scale evaluates perceived therapeutic changes according to the following values[24].

Hip Abductor Muscle Strength

Maximal isometric strength of the hip abductor muscles was assessed using a handheld digital dynamometer and recorded in Newtons. For this purpose, the participant lay in a side-lying position, with the limb to be tested positioned on top and aligned horizontally. In this position, the hip and knee of the lower limb (the limb positioned underneath) were flexed to 90 degrees. The participant then performed a maximal isometric contraction against the handheld dynamometer for 3 seconds, followed by a 30-second rest period[25]. Each measurement was repeated three times, and the mean of the three trials was normalized to body weight and recorded.

Flexibility of the Hip Abductor Muscles

Flexibility of the iliotibial band was measured using the Reese and Bandy technique and the Ober test. For this assessment, the participant lay in a side-lying position, and the hip and knee joints of the lower limb were flexed to 45 and 90 degrees, respectively, to stabilize the pelvis[26]. One examiner placed their right hand on the participant’s upper pelvis to stabilize it by applying downward pressure. Using the left hand, the same examiner passively positioned the hip joint of the upper limb into abduction and then extension, aligned with the trunk.

The participant was then asked to relax all lower-limb muscles and allow the upper limb to descend toward the table (hip adduction). As the limb descended, the examiner prevented hip flexion and internal rotation. During the test, the examiner maintained 90 degrees of knee flexion and ensured that no pelvic tilt or internal rotation and flexion of the hip occurred. In this position, a second examiner placed an inclinometer on the distal third of the participant’s thigh and recorded the degree of hip adduction. If the thigh dropped below the horizontal level, a positive value was recorded, and if it remained above the horizontal level, a negative value was recorded. Each measurement was repeated three times, and the mean of the three trials was recorded as the participant’s flexibility value.

**Strengthening Exercise Program**

Participants were instructed to refrain from running for four weeks and to avoid the use of analgesic or anti-inflammatory medications, except for acetaminophen (paracetamol) (25). Participants in both groups received a treatment protocol that included ice pack massage and strengthening exercises. Based on previous studies, participants were instructed to apply ice pack massage to the painful area twice daily for 10 minutes during the first two weeks [27].

In addition, participants received a strengthening exercise program consisting of:

1. side-lying hip abduction exercise,
2. clamshell exercise,
3. pelvic drop exercise,
4. single-leg step-down exercise,
5. forward and backward lunge exercise,
6. modified matrix exercise, and
7. wall banger exercise.

The strengthening program primarily emphasized the gluteus medius and gluteus maximus muscles (to improve lower-limb alignment relative to the trunk), as well as the hip external rotator muscles, based on evidence indicating that weakness of the hip external rotators is associated with increased hip internal rotation in runners [27-29].

Participants were instructed to follow a graded return-to-training protocol when resuming their athletic activities and to reduce training volume if pain was experienced. This protocol was recommended to facilitate a safe return to performance according to individual readiness. Graded exercise programs theoretically apply an appropriate level of stress to tissues, promoting tissue adaptation, optimal strength, and structural integrity, in accordance with Wolff’s law.

**Stretching and Foam Rolling Exercise Program**

Participants in the experimental group, in addition to the intervention provided to the control group, received an exercise program consisting of foam rolling and stretching exercises. The program was designed based on previous studies and included:

1. standing stretch,
2. pretzel stretch in a side-lying position, and
3. iliotibial band release using a foam roller.

Throughout the 8-week study period, attendance at all training sessions was recorded. The exercise program for both groups was conducted for 8 weeks, with each session lasting 25 to 30 minutes, three times per week. Training sessions included a 5-minute warm-up and a 5-minute cool-down. The 5-minute warm-up consisted of walking, marching in place, high knees, jogging, and selected mobility exercises. The intensity of the training sessions for both groups was monitored using the Borg Rating of Perceived Exertion scale.

4. **Statistical Analysis Method**

Data analysis was performed using SPSS software, version 23. Initially, the Shapiro–Wilk test was used to examine the normality of data distribution, and Levene’s test was applied to assess the homogeneity of variances between groups. Subsequently, analysis of covariance (ANCOVA) was conducted to evaluate the main and interaction effects of the study variables in testing the research hypotheses. All statistical analyses were performed at a significance level of p ≤ 0.05.

5. **References**

1.	Kakouris N, Yener N, Fong DT. A systematic review of running-related musculoskeletal injuries in runners. Journal of sport and health science. 2021;10(5):513-22.

2.	Khaund R, Flynn SH. Iliotibial band syndrome: a common source of knee pain. American family physician. 2005;71(8):1545-50.

3.	Baker RL, Fredericson M. Iliotibial band syndrome in runners: biomechanical implications and exercise interventions. Physical Medicine and Rehabilitation Clinics. 2016;27(1):53-77.

4.	Ellis R, Hing W, Reid D. Iliotibial band friction syndrome—a systematic review. Manual therapy. 2007;12(3):200-8.

5.	Hutchinson L, Lichtwark G, Willy R, Kelly L. The iliotibial band: a complex structure with versatile functions. Sports Medicine. 2022;52(5):995-1008.

6.	Balachandar V, Hampton M, Riaz O, Woods S. Iliotibial Band Friction Syndrome: A Systematic Review and Meta-analysis to evaluate lower-limb biomechanics and conservative treatment. Muscles, Ligaments & Tendons Journal (MLTJ). 2019;9(2).

7.	Fairclough J, Hayashi K, Toumi H, Lyons K, Bydder G, Phillips N, et al. The functional anatomy of the iliotibial band during flexion and extension of the knee: implications for understanding iliotibial band syndrome. Journal of anatomy. 2006;208(3):309-16.

8.	Orchard JW, Fricker PA, Abud AT, Mason BR. Biomechanics of iliotibial band friction syndrome in runners. The American journal of sports medicine. 1996;24(3):375-9.

9.	Park JJ, Lee HS, Kim J-H. Effect of acute self-myofascial release on pain and exercise performance for cycling club members with iliotibial band friction syndrome. International Journal of Environmental Research and Public Health. 2022;19(23):15993.

10.	Everhart JS, Kirven JC, Higgins J, Hair A, Chaudhari AA, Flanigan DC. The relationship between lateral epicondyle morphology and iliotibial band friction syndrome: A matched case–control study. The Knee. 2019;26(6):1198-203.

11.	Bolia IK, Gammons P, Scholten DJ, Weber AE, Waterman BR. Operative versus nonoperative management of distal iliotibial band syndrome—where do we stand? A systematic review. Arthroscopy, Sports Medicine, and Rehabilitation. 2020;2(4):e399-e415.

12.	Friede MC, Innerhofer G, Fink C, Alegre LM, Csapo R. Conservative treatment of iliotibial band syndrome in runners: Are we targeting the right goals? Physical Therapy in Sport. 2022;54:44-52.

13.	Beals C, Flanigan D. A review of treatments for iliotibial band syndrome in the athletic population. Journal of sports medicine. 2013;2013.

14.	Foch E, Brindle RA, Pohl MB. Lower extremity kinematics during running and hip abductor strength in iliotibial band syndrome: A systematic review and meta-analysis. Gait & Posture. 2023.

15.	Louw M, Deary C. The biomechanical variables involved in the aetiology of iliotibial band syndrome in distance runners–a systematic review of the literature. Physical Therapy in sport. 2014;15(1):64-75.

16.	Watcharakhueankhan P, Chapman G, Sinsurin K, Jaysrichai T, Richards J. The immediate effects of Kinesio Taping on running biomechanics, muscle activity, and perceived changes in comfort, stability and running performance in healthy runners, and the implications to the management of Iliotibial band syndrome. Gait & Posture. 2022;91:179-85.

17.	Behm DG, Wilke J. Do self-myofascial release devices release myofascia? Rolling mechanisms: a narrative review. Sports Medicine. 2019;49(8):1173-81.

18.	Nguyen AP, Detrembleur C, Van Cant J. Conservative treatment for iliotibial band syndrome: Are we facing a research gap? A scoping review of 98 studies with clinical perspectives. Physical Therapy in Sport. 2023.

19.	Miccio S, Berardi A, Tofani M, Galeoto G. Conservative Rehabilitation Treatments of Iliotibial Band Syndrome: A Systematic Review. Muscles, Ligaments & Tendons Journal (MLTJ). 2021;11(1).

20.	Cruz-Díaz D, Kim K-M, Hita-Contreras F, Bergamin M, Aibar-Almazán A, Martínez-Amat A. Effects of 12 weeks of Tai Chi intervention in patients with chronic ankle instability: a randomized controlled trial. Journal of sport rehabilitation. 2020;29(3):326-31.

21.	Katz J, Melzack R. Measurement of pain. Surgical Clinics of North America. 1999;79(2):231-52.

22.	Rohani C, Abedi HA, Langius A. The Iranian SF-12 Health Survey version 2 (SF-12v2): Factorial and convergent validity, internal Consistency and test-retest in a healthy sample. Iranian Rehabilitation Journal. 2010;8(2):4-14.

23.	Binkley JM, Stratford PW, Lott SA, Riddle DL, Network NAORR. The Lower Extremity Functional Scale (LEFS): scale development, measurement properties, and clinical application. Physical therapy. 1999;79(4):371-83.

24.	Kamper SJ, Maher CG, Mackay G. Global rating of change scales: a review of strengths and weaknesses and considerations for design. Journal of Manual & Manipulative Therapy. 2009;17(3):163-70.

25.	Beddows TP, van Klij P, Agricola R, Tak IJ, Piscaer T, Verhaar JA, et al. Normal values for hip muscle strength and range of motion in elite, sub-elite and amateur male field hockey players. Physical Therapy in Sport. 2020;46:169-76.

26.	Reese NB, Bandy WD. Use of an inclinometer to measure flexibility of the iliotibial band using the Ober test and the modified Ober test: differences in magnitude and reliability of measurements. Journal of Orthopaedic & Sports Physical Therapy. 2003;33(6):326-30.

27.	Fredericson M, Weir A. Practical management of iliotibial band friction syndrome in runners. Clinical Journal of Sport Medicine. 2006;16(3):261-8.

28.	Friede MC, Klauser A, Fink C, Csapo R. Stiffness of the iliotibial band and associated muscles in runner’s knee: Assessing the effects of physiotherapy through ultrasound shear wave elastography. Physical Therapy in Sport. 2020;45:126-34.

29.	McKay J, Maffulli N, Aicale R, Taunton J. Iliotibial band syndrome rehabilitation in female runners: a pilot randomized study. Journal of orthopaedic surgery and research. 2020;15(1):1-8.

**Declaration of Commitment**

I, .......**Aynollha naderi**......, hereby commit that I will not initiate my research before obtaining the ethics approval from the Biomedical Research Ethics Committee at Shahrood University of Technology. I also pledge to complete all ethics application forms honestly and in accordance with the ethical guidelines of the Ministry of Science, Research, and Technology and the Ministry of Health and Medical Education. Furthermore, I will adhere to ethical principles in the preparation of scientific work and take all necessary measures to prevent any research misconduct.

**Date, Name, and Signature of Principal Researcher (Supervisor or Project Investigator):**
